# Interrogation of global mutagenesis data with a genome scale model of *Neisseria meningitidis *to assess gene fitness *in vitro *and in sera

**DOI:** 10.1186/gb-2011-12-12-r127

**Published:** 2011-12-30

**Authors:** Tom A Mendum, Jane Newcombe, Ahmad A Mannan, Andrzej M Kierzek, Johnjoe McFadden

**Affiliations:** 1 Faculty of Health and Medical Sciences, University of Surrey, Guildford, GU2 7XH, UK

## Abstract

**Background:**

*Neisseria meningitidis *is an important human commensal and pathogen that causes several thousand deaths each year, mostly in young children. How the pathogen replicates and causes disease in the host is largely unknown, particularly the role of metabolism in colonization and disease. Completed genome sequences are available for several strains but our understanding of how these data relate to phenotype remains limited.

**Results:**

To investigate the metabolism of *N. meningitidis *we generated and then selected a representative Tn*5 *library on rich medium, a minimal defined medium and in human serum to identify genes essential for growth under these conditions. To relate these data to a systems-wide understanding of the pathogen's biology we constructed a genome-scale metabolic network: Nmb_iTM560. This model was able to distinguish essential and non-essential genes as predicted by the global mutagenesis. These essentiality data, the library and the Nmb_iTM560 model are powerful and widely applicable resources for the study of meningococcal metabolism and physiology. We demonstrate the utility of these resources by predicting and demonstrating metabolic requirements on minimal medium, such as a requirement for phosphoenolpyruvate carboxylase, and by describing the nutritional and biochemical status of *N. meningitidis *when grown in serum, including a requirement for both the synthesis and transport of amino acids.

**Conclusions:**

This study describes the application of a genome scale transposon library combined with an experimentally validated genome-scale metabolic network of *N*. *meningitidis *to identify essential genes and provide novel insight into the pathogen's metabolism both *in vitro *and during infection.

## Background

*Neisseria meningitidis *is an obligate commensal of the human nasopharynx, which in most cases leads to an asymptomatic infection. However, on rare occasions the bacteria cross the mucosal barrier to cause a bacteremia that can progress rapidly to life threatening septicemia and/or meningitis. Although many virulence factors involved in pathogenesis have been identified, a neglected aspect of meningococcal virulence is the metabolic adaptations required to survive and proliferate in the host. Metabolism is known to play a key role in host-pathogen interactions in both acute and persistent infections. A genome-wide analysis of virulence genes required for systemic meningococcal infection in the rat showed that approximately half were involved in metabolism and nutrient transport [1]. Mutation of genes involved in lactate assimilation [2,3], glutamate assimilation [4,5] iron uptake [6], and sugar fermentation [7] all result in *N. meningitidis *strains that were attenuated in mice and rat models. Yet, many questions remain, such as the nature of carbon and nitrogen substrates utilized at different body sites and the metabolic pathways required for infection.

Transposon libraries are a valuable resource with which to identify gene function on a genome-wide scale [8-12]. Using PCR-based amplification methods, such as transposon site hybridization (TraSH) [8,9], microarray tracking of transposon mutants [10] or genomic array footprinting [11], in combination with either microarrays or high-throughput sequencing [12], transposon libraries can be rapidly screened to identify mutants with fitness deficits and thereby probe the pathogenesis and physiology of the target organism. Transposon libraries have been constructed in *N. meningitidis *and used to identify genes involved in several functions, including competence [13], binding of complement proteins [14], polymyxin B sensitivity [15], and survival on human nasopharynx explants [16]. In addition, a partial library of defined mutants has recently been constructed, consisting of some 796 mutants (estimated to represent 57% of the non-essential genes [17]). However, all these libraries are relatively small, and cannot therefore be used to provide genome-wide scans of gene function. Here we generated a large and representative transposon library of *N. meningitidis*. Using a validated PCR-based amplification protocol (TraSH), the fitness effect of mutating every gene within the library was assessed during growth on a complete media, a minimal media (MM) and in 100% complement inactivated serum.

The interpretation of such genome-scale datasets to determine the role and function of genes in terms of the overall biology of the pathogen remains difficult, as we lack a system-level understanding of gene function. A key tool that has provided such system-level insight into gene function for many other micro-organisms is the genome-scale metabolic network [18-22]. Such models can be used to predict growth yields, nutritional requirements or gene essentiality under a variety of conditions, which can then be compared to experimental data to test and refine the network. A metabolic network has previously been constructed for *N. meningitidis *[21], which was used to optimize media composition. However, the model was not genome-scale and cannot therefore provide system-wide analysis of metabolic capability. We here describe the construction of a genome-scale metabolic network, Nmb_iTM560, for *N. meningitidis*. However, an essential step in the construction of any biological model is its interrogation with experimental data. To this end we compared the Nmb_iTM560 output with data from the library selections. The results were used to improve the model and provide novel system-wide insights into meningococcal metabolism both *in vitro *and *ex vivo*. 

## Results

### The Neisserial Genome Scale model, Nmb_iTM560

The starting point for Nmb_iTM560 was the *i*AF1260 model of *Escherichia coli *K12 [22]. A preliminary model was generated by mapping orthologous *N. meningitidis *MC58 genes onto the model, while reactions encoded by genes with no *N. meningitidis *orthologues were removed so long as a solution - that is, biomass production - remained feasible. The model was then refined by the addition of *N. meningitidis *specific pathways and genes. Orphan reactions were minimized; 'dead end' reactions (those that contain a metabolite that is either only consumed or only produced ) were assessed, and elemental stoichometries were balanced. The completed Nmb_iTM560 model consists of 1,255 reactions, encoded by 586 genes and 59 orphan genes (where an essential or documented function is not associated with an annotated gene). These represent 65% of the reactions in the original *E. coli *K12 *i*AF1260 model. The model is available online [23] and in Additional file 1. Nmb_iTM560 retains many of the features of the *i*AF1260 *E. coli *model on which it is based, including extracellular, periplasmic, and cytoplamic compartments; a minimum number of grouped reactions; and many of the ΔG^0 ^based reversibility predictions. Reactions were added to complete pathways to alanine (alanine-pyruvate transaminase), biotin (pimeloyl-CoA synthase), heme (protoporphyrinogen oxidase), serine (phosphoglycerate dehydrogenase), ethanol (aldehyde dehydrogenase) [19,21], thiamine synthesis and for the reduction of thiosulfate [24]. Several pathways not found in *E. coli *were also added, such as those for asparagine biosynthesis via aminoacyl tRNA, meningococcal lipopolysaccaride (LPS) synthesis, capsule synthesis, and iron acquisition from ferric ions, hemoglobin, transferrin and lactoferrin. Finally, the biomass equation was modified to reflect the neisserial cell composition (Additional file 2) [19,25,26]. Three biomass equations were formulated: a wild type biomass for determining fluxes, and so on; a biomass lacking non-essential cellular components such as LPS, capsule, and so on, so that reaction and gene essentiality could be assessed; and a biomass minus capsule for comparison to the model of Baart *et al *[21]. Cofactor requirements were incorporated into reaction equations as described elsewhere [20]. A P/O ratio of 1.33 and ATP maintenance requirements were taken from Baart *et al *[19].

Using flux balance analysis (FBA), the Nmb_iTM560 model predicts growth of *N. meningitidis *on minimal media with a range of carbon sources, including glucose, lactate, pyruvate, and some amino acids, including glutamate, but not on acetate. Yields were comparable to predictions from the neisserial model constructed by Baart *et al *[21] (Table 1). Growth in sera was modeled by opening substrate gates for metabolites present in sera. Using flux variable analysis (FVA), the model predicted maximal growth rates in sera utilizing either glucose, lactate, or α-ketoglutarate as its primary carbon source (Additional file 3). Gene essentiality data from the model were used to refine the interpretation of TraSH data (Additional file 4).

**Table 1:** Predicted Nmb_iTM560 biomass yields

Substrate	Nmb_iTM560 yield	Yield reported by Baart
Glucose	11.4	10.7
Lactate	9.8	9.6
Alpha-ketoglutarate	8.9	ND
Pyruvate	8.0	ND
Glutamate	9.9	8.2
Acetate	0	0

Nmb_iTM560 was used to determine biomass yields (g biomass molC^-1^) when grown on minimal media with various carbon sources. Values are compared to those reported by Baart *et al *[21]. ND not determined.

### Transposon mutant library construction and initial selection

A library consisting of approximately 14,000 Tn*5 *insertional knockouts was constructed in the group C strain *N. meniningtidis *L91543 [27], a strain chosen because of its high transformation efficiency. The library was initially recovered on Columbia Agar Base supplemented with blood (CAB), a rich undefined media, to ensure that the library was as comprehensive as possible. Generating a genome-scale library directly on minimal media was not possible as the transformation efficiency when recovered on MM agar was too low to produce a representative library.

### TraSH analysis

**Figure 1:**
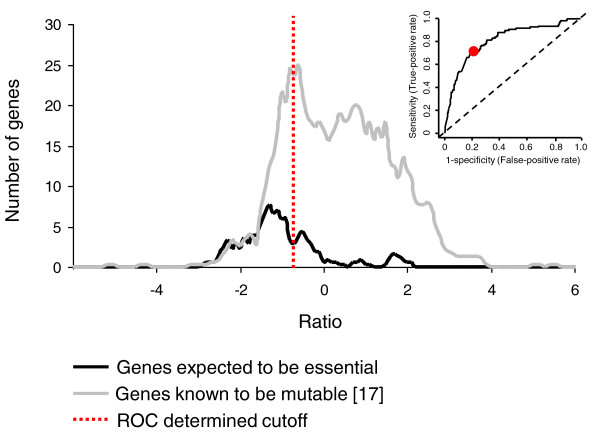
**Distribution of TraSH ratios and ROC curve**. The distribution of ratios during the initial selection of genes expected to be essential and those known to be non-essential [17]. The receiver operating characteristic (ROC) derived cutoff is indicated (red dotted line) and the ROC curve is shown in the inset. Values are the average of five separate library aliquots.

Using a modified version of the TraSH protocol described by Badarinarayana *et al*. [28], the genomic loci adjacent to the transposon insertion sites were amplified. These amplified fragments were hybridized to microarrays with labeled genomic DNA as a reference channel. The relative microarray signals were used to provide predictions of the abundance of mutants within the library. To establish a cutoff ratio that distinguished between essential and non-essential genes, we adopted an approach similar to that used by Sassetti *et al *[9]. Two gene lists were generated, one containing genes that were expected to be essential, the other containing genes expected to be non-essential. For the essential gene list we selected 110 neisserial orthologs of genes shown to be essential for survival in *E. coli *[29] when grown on a rich undefined media. For the non-essential gene list we used the 796 genes that have been shown to be mutable on rich GC agar by Rusniok *et al*. [17] (Additional file 5). Using receiver operating characteristic (ROC) [20] analysis with these datasets we established an optimum cutoff of -0.738 (Figure 1), which distinguished the two datasets with a sensitivity and specificity of 0.708 and 0.794, respectively (*P *= 3 × 10^-29^).

### TraSH validation

Because TraSH data represent a continuous measure of fitness within a competing pool of mutants, rather than a single binary character, an assessment of whether any single gene should be considered essential should be treated with some caution [29]. For example, severely attenuated mutants are likely to appear to be essential as they are either lost from the library during passage or become so rarefied as to give a TraSH ratio that resembles an essential gene. To determine the reliability and accuracy of our data, we validated the TraSH methodologies and the accuracy of the results.

First the data were assessed for bias with respect to gene length (Pearson's r = 0.008), which would imply inadequate library coverage, and operonic position (p = 0.71), but no significant correlations were found (Additional file 5).

To validate the methodologies, we generated a library of known mutants by combining DNA from 17 library isolates with Tn*5 *insertion loci that had been previously sequenced. This library was amplified and the data analyzed in the same manner as the original library to test the ability of our TraSH methodology to identify loci that contained inserts. ROC analysis was again used to establish a cutoff (using the 17 loci as one dataset and all the other genes as the other dataset), which distinguished the 17 loci with a sensitivity and specificity of 0.852 and 0.777, respectively, *P *= 2 × 10^-44 ^(Additional file 5).

To assess the accuracy of the TraSH data, we attempted to construct insertional knockouts in ten genes and compared the knockout phenotypes to the TraSH result. We tested eight genes that were predicted by TraSH to be non-essential on rich CAB agar but essential on MM agar, one gene (NMB1646) that was predicted to be non-essential on both media and one gene (*glnD*) that was predicted to be essential only on CAB. Knock-out mutants were successfully generated for all nine genes that were predicted by TraSH to be non-essential for growth on CAB. When phenotypes on MM agar were compared to TraSH predictions, only one was discordant (Table 2). No transformants were recovered for the *glnD *knockout on either MM agar or CAB; it is likely that this was due to the inherently low transformation efficiency of cells recovered on MM agar, and because it is indeed essential on CAB, although this could not be confirmed without further investigation. These data independently validate this TraSH methodology and demonstrate that it is able to identify essential and non-essential genes in this *N. meningitidis *library with a high degree of accuracy.

**Table 2:** Knock-out mutants, their TraSH ratios, phenotypic predictions and knockout phenotypes

			Initial growth on CAB			Growth on MM agar		
			
NMB	Function	Gene	TraSH prediction	TraSH ratio	Experimental phenotype	TraSH prediction	Change in TraSH ratio	Experimental phenotype
ΔNMB1646	Putative hemolysin III		Non-ess	0.74	Non-ess	Non-ess	1.40	Non-ess
ΔNMB0696	ABC transporter ATP-binding protein		Non-ess	0.495	Non-ess	Ess	-1.62	Non-ess
ΔNMB0719	Queuine tRNA-ribosyltransferase	*tgt*	Non-ess	1.25	Non-ess	Ess	-3.00	Ess
ΔNMB1338	Putative hydrolase		Non-ess	-0.034	Non-ess	Ess	-1.67	Ess
ΔNMB1277	Putative transporter		Non-ess	-0.0625	Non-ess	Ess	-1.62	Ess
ΔNMB1295	Formamidopyrimidine-DNA glycosylase	*mutM*	Non-ess	-0.174	Non-ess	Ess	-1.49	Ess
ΔNMB0318	Multidrug resistance protein A	*emrA*	Non-ess	2.28	Non-ess	Ess	-3.29	Ess
ΔNMB2061	Phosphoenolpyruvate carboxylase	*ppc*	Non-ess	1.52	Non-ess	Ess	-1.83	Ess
ΔNMB0951	Succinate dehydrogenase	*sdhB*	Non-ess	1.10	Non-ess	Ess	-3.13	Ess

A number of knockout mutants were generated by insertional inactivation and their ability to grow on Columbia Agar Base (CAB) and minimal media agar (MM) assessed. These knockout phenotypes were compared to TraSH predicted phenotypes. A TraSH ratio of less than -0.738 is indicative of essentiality in the initial selection. Due to low transformation efficiencies when plating transformants onto MM it was not possible to determine whether ΔNMB1203 was viable on MM. TraSH ratios are the average of five biological replicates (three for sera).

### *In vitro *gene essentiality on CAB medium

Genes without transposon insertions (defined as having a ratio of less than -0.738) in the initial library were deemed essential for growth of *N. meningitidis *in the rich blood-based CAB agar (Additional file 6). Using this cutoff, 585 genes, 26% of the genome, are essential. This compares with 302 essential genes in *E. coli *[29]. When the list of essential neisserial genes was compared to essential genes in other organisms, 52 to 59% had essential orthologs in *E. coli *[29,30], 52% had essential orthologs in *Salmonella typhi *[12] and 44% had essential orthologs in *Helicobacter pylori *[10].

**Figure 2:**
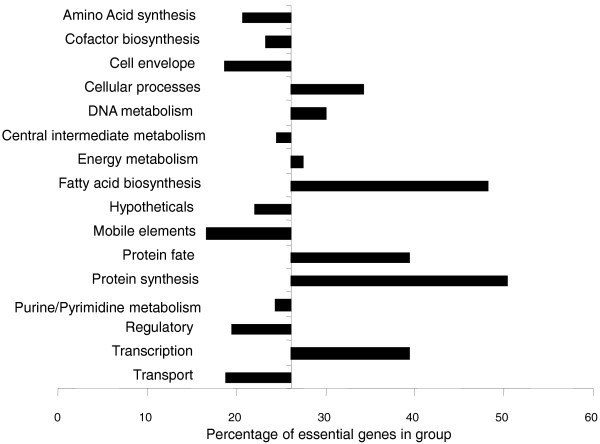
**Functional group essentiality**. The percentage of genes in each functional group shown to be essential by TraSH during the initial library selection. The origin is set to 26%, the percentage of essential genes in the whole genome. Cell envelope genes include those involved in peptidoglycan, capsule and lipopolysaccaride (LPS) synthesis as well as pili; cell processes includes genes associated with cell division, pathology, and so on; intermediate metabolism includes phosphate and sulfur metabolism; energy metabolism includes glycolysis, the pentose phosphate pathway, the tricarboxylic acid cycle, fermentation, electron transport and ATP synthesis; mobile elements includes prophage-, plasmid- and transposon-associated genes; other categories are self-explanatory.

**Figure 3:**
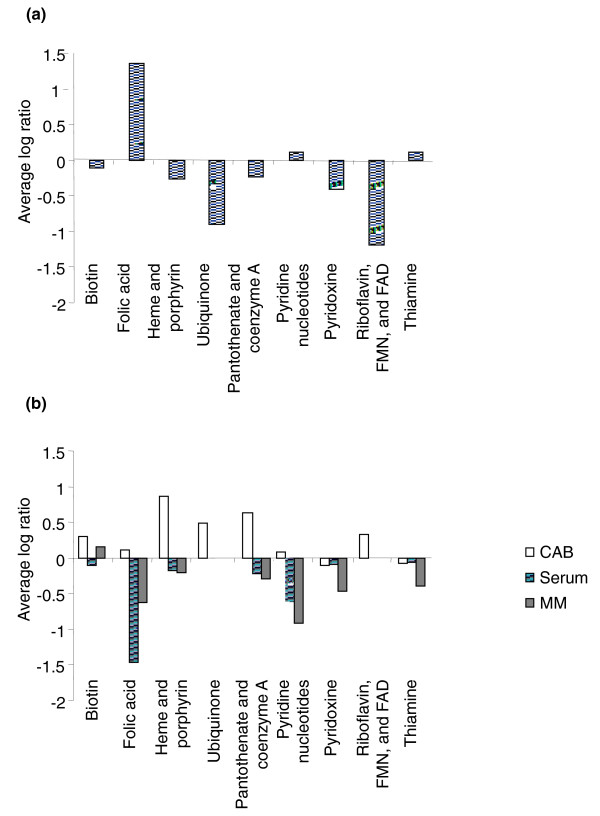
**TraSH ratios of cofactor biosynthetic gene groups**. **(a) **Average TraSH ratios from the initial selection for those cofactor biosynthetic genes predicted by Nmb_iTM560 to be essential. **(b) **Average competitive indices (the change in TraSH ratios) following sequential culturing on Columbia Agar Base (CAB, clear bars), serum (lined bars) and minimal media (MM) agar (filled bars) for those cofactor biosynthetic genes predicted by Nmb_iTM560 to be essential. Values are the average of five biological replicates (three for sera). All the Nmb_iTM560 predicted essential genes for ubiquinone and riboflavin synthesis were also predicted to be essential by TraSH during the initial selection and so are missing from the minimal media agar and serum selections.

Genes that are predicted to be essential on CAB were predominantly associated with processes such as transcription, protein synthesis, protein export and modification, DNA replication, and genes involved in fatty acid biosynthesis and cell wall synthesis (Figure 2). Only a few metabolic pathways were predicted to be essential, implying that the CAB agar provides a wide range of metabolic intermediates in sufficient quantities to complement most auxotrophic knockouts. Those that were essential included genes of the Entner-Doudoroff pathway, which is the primary route of sugar catabolism in *N. meningitidis *[19,31], and has been proposed as being essential in *N. meningitidis *[19]. The glycolytic genes and those of the tricarboxylic acid (TCA) cycle that further catabolize these metabolites were not essential, with the exception of those between glyceraldehyde-3-phosphate and phosphoenolpyruvate (assuming *gapB*, NMB2159 is uniquely catabolic). Of the genes involved in amino acid, purine, pyrimidine or cofactor biosynthesis (Figure 2), only a few pathways were predicted to be essential, notably those involved in riboflavin and siroheme (a cofactor involved in SO_4 _reduction) synthesis (Figure 3). Unexpectedly, the genes encoding a Na^+ ^translocating NADH-quinone reductase were essential, as were genes encoding components of the electron transport chain, such as ubiquinone synthesis, cytochrome *c *(*cytC, cyc*), cytochrome *c *biogenesis (*ccsA*), and cytochrome *c *oxidase (*ccoO, ccoP*). Genes for H^+ ^translocating NADH-quinone reductase and the cytochrome *bc1 *complex were not essential. Very surprisingly, 23 of the 45 genes (NMB0896-0919 and NMC0851-0897) of the prophage IHT-E [32] were predicted by TraSH to be essential. As the composition of CAB is undefined, comparisons between predictions of the Nmb_iTM560 model and TraSH data from these experiments were not possible.

Functional groups for which transposon insertion did not cause a loss of fitness on CAB agar included those involved in transport (again indicating a level of redundancy in nutrient availability), regulation, pilus biosynthesis and modification, DNA transformation, pathogenesis, ATP synthase and mobile elements such as transposases (Figure 2). Only 21% of hypothetical genes were essential, although this still leaves 165 with ratios of less than -0.737, indicating that many functionally important genes still remain to be annotated.

To distinguish attenuated mutants from mutants with essential or neutral phenotypes, the initial library was sequentially passaged three times on CAB agar (approximately 60 divisions in total). By comparing ratios before and after this selection, a competitive index was generated, log_2_(ratio_subsequent selection_) - log_2_(ratio_initial selection_), that quantifies the change in the abundance of each mutant, that is, its relative fitness. Mutants with a low competitive index, and hence a lowered fitness, after sequentially culturing on CAB agar are likely to be attenuated on CAB agar, rather than essential [9,12]. Genes in this category included many TCA cycle and glycolytic genes, several genes involved in purine (and to a lesser extent pyrimidine) synthesis, transcription (Figure 2), translation (for example, *rplOQST*), DNA replication (for example, *recCG*) and several involved in cell division (*minCDE, ftsAEK*), some of which are known to give attenuated phenotypes when mutated in *N*. *gonorrhoeae *[33]. Mutations in a few genes appeared to provide a selective advantage for growth on CAB agar, that is, have a high competitive index, most notably genes involved in the synthesis of the outer core of LPS (*lgtA, pgmB, galU*).

### Identification of mutations with conditional fitness effects in minimal media and integration with the Nmb_iTM560 model

**Figure 4:**
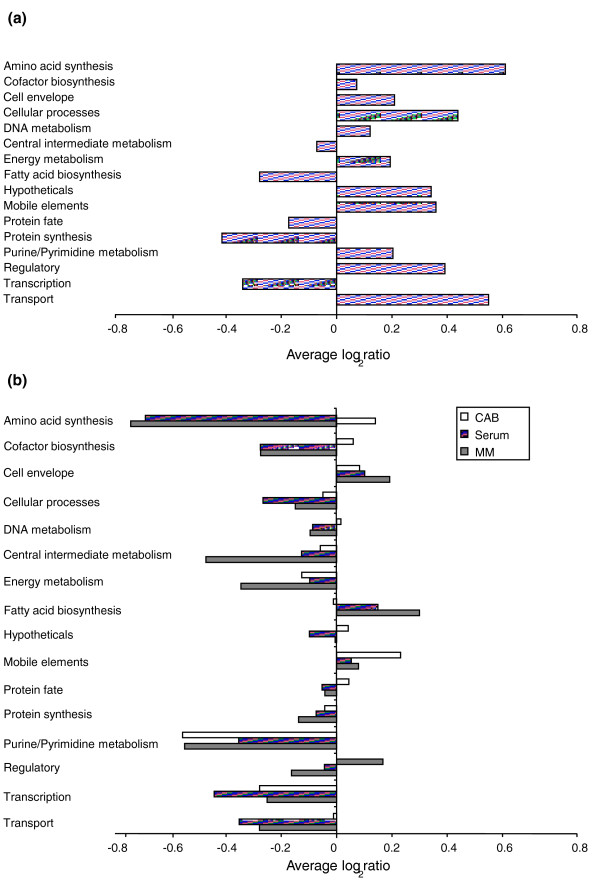
**TraSH ratios of functional gene groups. (a) **Average TraSH ratios for functional gene groups from the initial selection. **(b) **Average competitive indices (the change in TraSH ratios) for functional gene groups following sequential culturing on Columbia Agar Base (CBA; clear bars), serum (lined bars) and minimal media (MM) agar (filled bars). All values are the average of five biological replicates (three for sera). Cell envelope genes include those involved in peptidoglycan, capsule and lipopolysaccaride (LPS) synthesis as well as pili; cell processes includes genes associated with cell division, pathology, and so on; intermediate metabolism includes phosphate and sulfur metabolism; energy metabolism includes glycolysis, the pentose phosphate pathway, the tricarboxylic acid cycle, fermentation, electron transport and ATP synthesis; mobile elements includes prophage, plasmid and transposon associated genes; other categories are self-explanatory.

**Figure 5:**
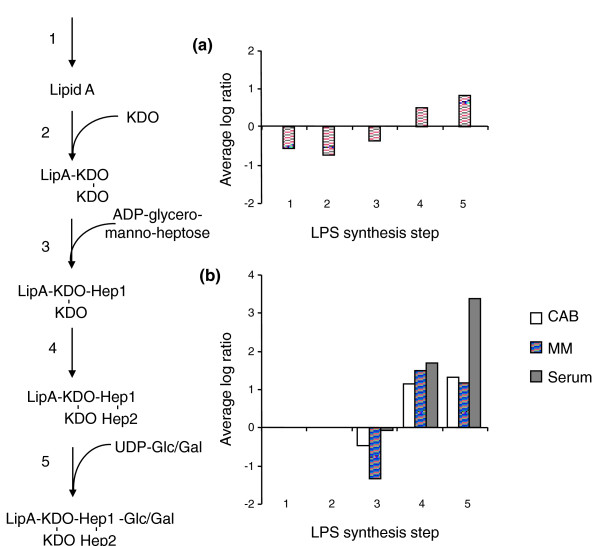
**TraSH ratios of lipopolysaccaride synthesis gene groups**. **(a) **Average TraSH ratios from the initial selection for genes involved in lipopolysaccaride (LPS) synthesis. **(b) **Average competitive indices (the change in TraSH ratios) following sequential culturing on Columbia Agar Base (CAB; clear bars), serum (lined bars) and minimal media (MM) agar (filled bars) for genes involved in LPS synthesis. Values are the average of five biological replicates (three for sera). The pathway for LPS synthesis [58] is shown on the left. KDO, 2-keto-3-deoxyoctonoic acid.

To identify mutants that lose fitness on minimal media, the initial library was sequentially passaged three times on MM agar (approximately 60 divisions in total), containing glucose as the sole carbon source, NH_4 _as the sole nitrogen source, and SO_4 _and thiosulfate as sulfur sources. A competitive index was again calculated and those mutations that conferred a large fitness cost were considered to be 'conditionally essential' genes - that is, they became essential only when *N. meningitidis *was transferred from nutrient-rich CAB medium to MM agar. It is important to note that this group of genes is distinct from the genes that are essential for growth on MM agar, which would also include many mutations lost from the initial library. Many of these conditionally essential genes were involved in amino acid, purine/pyrimidine synthesis and cofactor synthesis; all presumably nutrients available in CAB medium that on MM are required to be synthesized (Figure 4). Other functional groups that contained conditionally essential genes included central carbon metabolism (pyruvate metabolism, TCA, and so on), intermediary metabolism (particularly nitrogen and sulfur acquisition) and several nutrient transport genes, such as those involved in the transport of heavy metals, iron (*fbpAB*, but not *fbpC *[34]), sulfate and zinc. A number of efflux systems, such as *emrAB *(*emrA *was confirmed by gene knockout (Table 2)) and *mtrFC*, also lost fitness as did components of the *tonB *system, which normally is involved in the transport of non-ferric iron. Less predictable was the loss of fitness of several genes associated with DNA repair, and genes associated with oxidative stress (*bfrAB, sodC, dsbB*). Once again, mutations involved in outer core LPS synthesis (*lgtABF, galE, lgt*) resulted in a fitness advantage (Figure 5).

To evaluate the accuracy of the Nmb_iTM560 model, we tested its ability to predict MM conditionally essential genes. We first performed FBA to identify all genes predicted to be essential on MM (Additional file 4). Any gene that had already been identified by TraSH as being essential during the initial selection was then removed from the analysis. The remainder were designated the Nmb_iTM560-predicted conditionally essential genes, which were compared with the experimentally determined conditionally essential genes. The Nmb_iTM560 model distinguished the TraSH determined essential gene from non-essential genes with an accuracy of 58% (*P *= 2 × 10^-4^; Additional file 4). For well-defined areas of metabolism, such as amino acid or purine/pyrimidine biosynthesis, data from the model and from TraSH correlated better, with 74 to 76% accuracy, indicating these pathways are well annotated in the model.

### Identification of mutations with fitness effects in human serum

The metabolic requirements for *N. meningitidis *growth in 100% human serum are likely to be similar to those for growth in blood, a function that is vital for meningococcal pathology. To provide insights into this *in vivo *metabolism, we sequentially cultured the transposon library three times in serum (approximately 40 divisions in total). To ensure that genes other than those involved in modulating the complement-mediated killing of meningococci could be identified, the serum was first complement inactivated. As with the MM agar selection, a competitive index was used to assess which genes were conditionally essential. The major groups of genes that became conditionally essential for growth in serum were: those involved in amino acid production, particularly those involved in the synthesis of aromatic amino acids (*trpBCE, aroCDGK*), leucine (*leuBC, ilvC*), histidine (*hisCG*), glycine (*glyA*) and proline (*proC, putA*); genes involved in the synthesis of purines but not pyrimidines, a phenomena that has previously been recognized in *E. coli *and *Salmonella enterica *[35]; and some cofactor genes, including those involved in folic acid, pantothenate and pyridine synthesis (Figures 3 and 4). It appears that serum does not provide these nutrients in sufficient quantities to support maximal growth. Mutations in many nutrient transport reactions also caused a loss of fitness, particularly genes required for iron acquisition (*exbBD, tonB, fetC, fbpB, lbpA*), a number of amino acid transporters (NMB0787, NMB0788, NMB2031), and lactate permease (NMB0543), a gene known to be required for fitness when *N. meningitidis *is grown on nasopharyngeal explants [2]. Several genes of central carbon metabolism (parts of TCA and pyruvate metabolism) were also conditionally essential.

Mutations that lost fitness in MM agar but not in sera include those involved in sulfur acquisition, suggesting that sulfur can be acquired from organic sources in sera, and H^+ ^translocating NADH dehydrogenase. Mutations that affect the surface structures of the cell again had fitness effects, particularly those involved in LPS synthesis. As with CAB medium, genes involved in synthesis of the LPS outer core, *lgtA, galU, galE *and *pgm*, had a growth advantage (Figure 5), while those involved in the synthesis of the inner core structure of LPS lost fitness (notably *gmhAB, rfaD, lgtF*, and so on).

As with the MM, we again performed FBA to identify all genes predicted by the model to be essential in sera and compared these to the experimental TraSH data. The Nmb_iTM560 distinguished TraSH-determined essential genes from non-essential genes in sera with an accuracy of 59% (*P *= 0.06; Additional file 4).

## Discussion

The combination of experimental data from TraSH and predictions from the genome-scale model using FBA (predicts maximal flux to biomass for knockouts or with different substrates) or FVA (predicts the range of flux for each reaction that is compatible with maximal growth) creates the potential for far more powerful insights into *N. meningitidis *metabolism than can be inferred from either the TraSH data or the model output alone.

### Describing *N. meningitidis *biochemistry - an example

The TCA cycle provides an example of how integrating the model and the TraSH data can be used to describe metabolic roles. Both the model and TraSH predict that much of the TCA cycle is required for growth on MM. However, Nmb_iTM560 predicts that this is due to a requirement for synthesis of TCA metabolic precursors, rather than catabolism, as maximal growth is feasible with just the flux required to meet the anabolic demand (Additional file 3). This provides an explanation for why TCA enzymes are not essential when these precursors are supplied by rich media such as CAB. As *N. meningitidis *has no glyoxalate shunt, anaplerotic replenishment of TCA cycle intermediates must be accomplished by another route: the model predicts that this is via PEP carboxylase operating in the carboxylating direction (Additional file 3). This result also provides an explanation for the requirement of *N. meningitidis *for elevated levels of CO_2 _when grown on MM agar and for the essentiality of PEP carboxylase on MM agar (Additional file 6, 7 and Table 2), both of which can be complemented by the addition of glutamate to the MM agar, which can be used to replenish the TCA cycle via α-ketoglutarate. The essentiality of PEP carboxylase also indicates that, in contrast to the situation in some other organisms, malate oxidoreductase (NMB0671) cannot function in the carboxylating direction in *N. meningitidis *as Nmb_iTM560 predicts this would complement PEP carboxylase.

### What can discrepancies between the model and TraSH tell us?

The Nmb_iTM560 model predicts the experimental TraSH data for growth on MM agar, particularly for genes involved in amino acid and purine/pyrimidine synthesis pathways, indicating that our knowledge of these pathways is reasonably accurate. However, some other pathways did not correlate well, suggesting incomplete gene annotations, gene regulatory effects or an incomplete understanding of the physiological role of the gene's function. An example of such genes are the Na^+ ^and H^+ ^translocating NADH reductases. *N. meningitidis*, in common with several other pathogens, encodes two translocating NADH dehydrogenases, one that translocates sodium and the other that translocates protons [36]. TraSH analysis shows that the genes encoding the Na^+^-translocating NADH dehydrogenase complex are essential for growth on CAB, but not the genes for the H^+ ^translocating NADH reductases. The functional reason for this is unclear. The translocating NADH reductases carry out several functions: the generation of electrochemical gradients; the supply of electrons to the electron transport chain; and the regeneration of NAD. The Nmb_iTM560 model predicts that all these functions are essential but that they can be performed by either Na^+ ^or H^+ ^translocating NADH reductase. As demonstrated here and elsewhere, the actual physiological role of Na^+ ^translocating NADH-quinone reductases in pathogens, and the reasons for its essentiality, remain poorly understood [36]. It has been proposed that it is primarily associated with its role in supplying electrons to the electron transport chain [37] and indeed much of the electron transport chain is also predicted by TraSH to be essential on CAB agar. On MM agar but not in serum, TraSH demonstrated that H^+ ^translocating NADH-quinone reductase becomes conditionally essential; again, it is unclear what function performed by this enzyme cannot be performed by the Na^+ ^translocating enzyme in combination with an H^+^/Na^+ ^antiport.

Another group of genes that generate discordant predictions are those with paralogs: the model predicts non-essentiality due to their apparent redundancy, and yet many are experimentally essential. The most likely explanation is that the paralogs have distinct and non-complementary functions. For example, the *N. meningitidis *genome encodes two paralogous genes annotated as glyceraldehyde-3P dehydrogenase, NMB2159 and NMB0207. However, the TraSH (Additional files 6 and 7) data indicates that only one of these, NMB2159, is essential for growth on CAB. This is consistent with previous studies that show NMB2159 to be the major glycolytic enzyme [38], while NMB0207 is involved in gluconeogenesis. Several other genes are also known to have divergent roles such as *dsbA1, dsbA2 *and *dsbA3 *[39-41], or *pilT-1 *and *pilT-2 *[42], and once again TraSH predicts dissimilar mutant phenotypes when selected on these media. By looking for other paralogs with dissimilar phenotypes (TraSH ratios), we can suggest divergent roles for *ackA, acpP, dnaQ, fabF, ftsK, ligA *and *potD*. Results such as these can be used to refine the Nmb_iTM560 model to more accurately reflect the biology of *N. meningitidis*.

Another potential source of discrepancy may be genetic differences between the L91543 strain in which the Tn*5 *library was constructed and the sequenced MC58 strain on which the *in silico *model was based. For example, the cys*DGHIJN *operon, which is involved in SO_4 _acquisition, is predicted to be redundant in Nmb_iTM560, as the operon is duplicated in the MC58 genome. However, the genes were essential by TraSH. It is possible that only one functional copy of the operon is present in L91543.

### Inferring neisserial physiology *in sera *

By carrying out TraSH analysis of the library when grown in serum and comparing the results to *in silico *simulations, we were able to identify metabolic function required for growth in sera. Sera contains a range of substrates, including glucose, lactate, α-ketoglutarate, low levels of amino acids, ions and transferrin [43], all substrates that Nmb_iTM560 predicts are capable of supporting growth. Because multiple carbon substrates are utilized, the model predicts that most of the genes of central carbon metabolism are not essential for growth in sera, the only exception being the genes encoding pyruvate metabolism, and those of the portion of the TCA cycle between succinate and malate. The TraSH data confirms these predictions.

One of the more surprising results of the TraSH experiments was the conditional essentiality in serum of a number of genes associated with amino acid synthesis. A requirement for aromatic amino acid synthesis when grown in sera has been observed previously in *S. typhimurium *[44] and has been associated with virulence in *N. meningitidis *[1]. Other amino acid synthesis genes with low TraSH ratios included those involved in the synthesis of leucine, histidine, glycine and proline. Some of these genes may be essential due to regulatory networks, as amino acid metabolism is tightly linked to wider physiological effects, and indeed several regulatory genes are also conditionally essential on MM agar (*lrp *and NMB0737). However, Nmb_iTM560 predicts that, for maximal growth, both amino acid synthesis and the assimilation of amino acids from sera are required (Additional file 4). This was consistent with the TraSH data, which showed that inactivation of either amino acid synthesis genes or amino acid transport genes caused loss of fitness in serum (Additional files 6 and 7). These conclusions are supported by recent transcriptomics data from *N. meningitidis *grown in blood, which identified many of these conditionally essential amino acid biosynthesis and transport genes as upregulated in blood culture [45].

Unfortunately the model does not include cofactor transport as these processes are not annotated and remain undefined. However, the TraSH data demonstrated that heme, pyridoxine and thiamine are present in serum in sufficient quantity to complement any auxotrophy. In contrast, biosynthesis of folic acid, pyridine and pantothenate was required for growth in serum. TraSH data also show that biotin was available in serum, although it also appears to be available in MM agar. Studies with *E. coli *have also demonstrated unexplained and apparently false non-essentiality for biotin biosynthesis genes [36] and so its availability in serum is difficult to assess.

Lastly, an interesting finding that is pertinent to meningococcal pathogenesis was the predicted increase in fitness of mutants that generate truncated LPS structures, a phenotype observed on all media and serum. Recent evidence indicates that *N. meningitidis *MC58 alters its LPS from the extended L3 immunotype to the truncated L8 immunotype [46] when grown on blood. Our TraSH data indicate that such a change would confer a fitness advantage on *N. meningitidis *that is independent of resistance to complement (Figure 5)

## Conclusions

We assessed global gene essentiality for *N. meningitidis *under a variety of growth conditions to provide a system-wide interrogation of gene function for this important pathogen. The transposon library constructed in this work and the associated TraSH data are valuable resources with which to investigate neisserial biology. Additionally, we constructed the first genome-scale metabolic model of *N. meningitidis *as a means to analyze and interrogate these and other system-wide, high-throughput data generated by, for example, transposon mutagenesis, transcriptomics, proteomics or metabolomics studies. Integrating the data from these two approaches we were able to validate the model and generate insights into gene function and the metabolic adaptations that *N. meningitidis *undergoes when grown on MM agar and in serum and so begin to examine the often neglected role that metabolism plays in neisserial pathogenesis. These data, the methods and the model are valuable, useful and widely applicable tools. The library and the model are publicly available [23].

## Materials and methods

### Strain and media

*N. meningitidis *L91543, a group C:2a:P1.2 organism, was used throughout. The organism was routinely cultured in CAB (Oxoid, Basingstoke, UK) supplemented with 6% defibrinated horse blood at 35°C with 5% CO_2_. MM agar [21] consisted of 31.1 mM glucose, 23.5 mM NH_4_Cl, 102 mM NaCl, 2.43 mM MgSO_4_.7H_2_O, 12.5 mM K_2_HPO_4_, 4.58 mM KH_2_PO_4_, 0.3 mM FeCl_3_.6H_2_O, 0.38 mM Na_2_S_2_O_3_.5H_2_O and 1.5% agar, as well as trace elements containing Ca^2+^, Zn^2+^, MoO_4_^2+^, Mn^2+^, Co^2+ ^and Cu^2+^. Erythromycin and kanamycin were used at 2 μg ml^-1 ^and 100 μg ml^-1^, respectively. MM supplements of glutamate were used at 1 mM. Pooled human serum (Sigma, Poole, UK) was complement denatured at 56°C for 30 minutes prior to use as growth medium.

### Library construction

DNA was extracted from *N. meningitidis *L91543 using a G-100 column (Qiagen, Crawley, UK) and 40 μg transposed *in vitro *with Tn*5 *containing an erythromycin resistance cassette from 13 μg pRC821 using 5 μl EZ-Tn*5*™ (Epicentre Biotechnologies, Cambridge, UK) and then phenol extracted. Transposed DNA was mixed with 25 U T4 DNA polymerase, 250 μm dNTPs and incubated at 37°C for 20 minutes, followed by 75°C for 10 minutes. The mix was precipitated and ligated with 10-15 U T4 DNA ligase for 180 minutes at 21°C and finally precipitated for use in transformations. *N. meningitidis *L91543 cultures were transformed with transposed DNA by mixing 0.5 μg transposed DNA with 10 μl of a suspension of *N. meningitidis *grown on CAB for 18 h and resuspended at an OD_600 _of 0.5. Mixes were dried onto CAB plates, incubated for 4 h and the resulting cells plated onto selective CAB agar.

### Library selection

DNA from initial selection was extracted from five separate aliquots of the frozen library. For experiments involving sequential passage, five aliquots (three for sera) of the library were sequentially subcultured three times for 24 h (48 h for MM agar) each on either CAB, MM agar or in 100% heat denatured serum (56°C for 30 minutes). DNA from each of these selected libraries was prepared using Qiagen G-100 columns, that is, five biological replicates. Three mock libraries were also produced to assess the fidelity of the TraSH amplification and microarray procedures. DNA from 17 isolates with pre-determined Tn*5 *insertion sites was extracted using Qiagen G-20 columns and mixed to give five replicate libraries of known composition containing equally amounts of DNA from each mutant.

### TraSH amplification

To amplify regions adjacent to transposons, a modified version of the protocol used by Badarinarayana *et al*. [28] was used. To avoid bias introduced by the position of restriction sites relative to Tn*5 *insertion sites, the DNA was sheared rather than restriction digested. DNA was sonicated three times for 30 s, pulsing for 1 s on and 1 s off, with a Vibracell VC300 with a CV18 sonicating tip (Sonics, Connecticut, USA) at 50% power to give lengths of approximately 500 bp. Sheared DNA (5 μg) was blunt ended using 5 U Klenow (Promega, Southampton, UK), 1 × Klenow buffer and 30 μM dNTPs for 15 minutes at 21°C. The enzyme was inactivated at 75°C for 20 minutes in the presence of 20 mM EDTA, and the DNA precipitated. Linker primers were annealed by mixing equimolar quantities in 10 mM Tris, 50 mM NaCl, pH 8, heating at 95°C for 2 minutes and allowing the mix to cool slowly. Annealed linkers (1 nmol) were ligated to 1 μg of the blunt-ended DNA with T4 DNA ligase at 14°C for 18 h, and isopropanol precipitated. Approximately 100 ng of ligated DNA was PCR amplified with primer T1.1 and either TR1 or TR3, reactions contained × 1 KOD buffer, 1.5 mM MgSO_4_, 0.2 mM dNTPs, 0.3 μM each primer and 0.02 U μl^-1 ^KOD enzyme (Novagen, Nottingham, UK). Reaction conditions were 95°C for 2 minutes, followed by 25 cycles of 95°C for 20 s, 65°C for 10 s and 70°C for 15 s. Products between 250 bp and 750 bp were gel extracted and a mix of TR1 and TR3 products were PCR amplified in a second PCR with primers T1.2, TR2 and TR4. Reactions contained × 1 KOD buffer, 1.5 mM MgSO_4_, 1 mM dCTP-Cy3, 0.2 mM dNTPs, 0.06 μM primer (Table 3), 0.02 U μl^-1 ^KOD and 20 ng of both the gel extracted T1.1/TR1 and the T1.1/TR3 first round PCR products. Reaction conditions were 95°C for 2 minutes, then 25 cycles of 95°C for 20 s, 65°C for 10 s, 70°C for 15 s, followed by 70°C for 5 minutes. Products were purified with MiniElute columns (Qiagen) and hybridized to microarrays.

**Table 3:** Primers used in this study

Linker 1	TAGGCGATCGGTTCAAGGCTGTGAGAACGTACGGAGACGGACGTAGCGTC
Linker 2	GACGCTACGTCCGGTCCTGGCTGACTC
T1.1	TAGGCGATCGGTTCAAGGCTGTGA
TR1	CTGGTATCTTTATAGTCCTGTCGGGTTTCG
TR3	TGCATCCCTTAACTTGTTTTTCGTGTACCTAT
T1.2	TGAGAACGTACGGAGACGGACGTAGC
TR2	ATTTGAGCGTCAGATTTCGTGATGCTTGTC
TR4	ATCGGTTCCGTACTATTTGGATCCGAGAC

Primers used to amplify and label genomic loci adjacent to transposons inserts.

### Microarrays and data analysis

A standard Cy5-labeled control channel sample was used as a standard in all the arrays. It was prepared by mixing 5 μg L91543 DNA with 15 μg random primers, heating to 95°C for 5 minutes and cooling on ice. The DNA was labeled by incubating at 37°C for 90 minutes in 1 × Klenow buffer, 100 μM dA/G/TTP, 20 μM dCTP, 80 μM dCTP-Cy5, 25 U Klenow. Reactions were cleaned with QIAquick PCR purification kit (Qiagen). PCR amplified transposon insertion loci labeled with Cy3 were mixed with this Cy5 labeled standard DNA to allow different selections to be compared, and hybridized to microarrays. The microarrays were supplied by BμGS [47]; each contained non-adjacent duplicate spots of PCR amplified probes for all open reading frames from *N. meningitidis *MC58, FAM18 and Z2491. Microarray slides were hybridized with control DNA (10 pmol Cy5) and TraSH amplified samples (5 to 10 pmol Cy3) as described by Stabler *et al*. [47]. Microarrays were scanned with a GenePix4000B scanner (Axon Instruments, Sunnyvale, USA). Data analysis was performed using R microarray package limma [48]. Spots labeled as bad or missing by GenePix Pro 6.0 were removed from the analysis. To ensure that only genes present in *N. meningitidis *L91543 were analyzed, spots with a control channel intensity of less than the background intensity plus 3.29 × standard deviations of the background (*P *< 0.001) were removed. So as not to remove genes that lack transposon insertions (essential genes), spots with low sample intensities of Cy3 were retained; where spot intensities were less than background intensity, the spot value was made to equal the background intensity. Duplicate spots were averaged and the resulting ratios normalized to the array median. Final values are the log_2 _of the average ratio of Cy3 to Cy5 from the five (three for sera) replicated libraries, that is, five biological replicates for each condition.

Genes represented on the array by more than one probe were assessed for relevance by comparing signals from the control channel (Cy5). Probes with a red fluorescence significantly lower than that of their orthologous partner were removed from the data.

To relate the mean ratios to predictions of the growth properties of each mutant, lists of genes with expected properties were compiled. A list of genes deemed as essential was derived from the Profiling of *E. coli *Chromosome database [49] of *E. coli *genes essential for growth on a rich undefined media containing beef extract, yeast extract, glucose and peptone [29]. *E. coli *genes that did not have an orthologous neisserial gene represented on the array were removed. To avoid genes whose essentiality may be predicated by genomic background or growth conditions, the list of essential genes included only essential genes involved in: peptidoglycan synthesis; cell division; DNA replication, recombination and repair; protein secretion and trafficking; ribosomal proteins; translation factors; tRNA aminoacylation; DNA-dependent RNA polymerase; RNA processing; and transcription factors. Finally, genes that are known to be mutable [17] were removed from the list. To determine a cutoff ratio for data from the initial selection, ROC analysis was carried out using these essential genes and the genes identified as mutable by Rusniok *et al*. [17], ratio distributions were determined and an optimum cutoff identified. Those genes with values below the cutoff were considered to be absent from the library and were removed from later analysis. Values for the secondary growth conditions were the difference between the initial ratio and the ratio from the secondary selection, to give a competitive index that quantified the change in mutant abundance when the library was moved between media. To determine whether there was a bias towards shorter genes being essential (having no transposon insertion), which would imply incomplete library coverage, ratios were compared to gene length using Pearson's correlation [10]. To assess bias for operonic position, operons were predicted [50], and the ratio of insertion in genes in operon position one was compared to the ratio of all the remaining genes with Student t test.

### Insertional gene inactivation

Chromosomal DNA from appropriate mutants of *N. meningitidis *8013 (kindly supplied by V Pelicic, Imperial College, London) was used to transform *N. meningitidis *L91543 as described previously. Cells were washed from the plate in water and plated onto CAB containing kanamycin.

### Nmb_iTM560 model construction

The genome-wide neisserial model was based on the *i*AF1260 model of *E. coli *K12 [22]. *E. coli *reactions with orthologous meningococcal genes were retained while those without orthologs were removed, an approach that has been successful elsewhere [51]. This initial model resulted in an unfeasible FBA solution, that is, zero flux to biomass. An alternative automated approach was designed, utilizing Cobra Toolbox [52], that generated a feasible prototype model containing the minimum number of non-orthologous reactions, which were then classified as orphan reactions. This initial neisserial model was modified with reference to Baart *et al*. [21], the Kyoto Encyclopedia of Genes and Genomes [53] and Biocyc [54,55] to include genes and reactions unique to *N. meningitidis*. The biomass composition was modified to reflect the neisserial cell composition using data for overall composition from Baart *et al*. [19] combined with details of phospholipids [25] and peptidoglycan [26] compositions. Finally, orphan reactions were again minimized, and checks made to ensure that 'dead-end' reactions were appropriate, and that elemental stoichiometries were balanced. Using FBA, gene and reaction essentiality scans were performed to identify knockouts that result in zero flux in the biomass reaction, that is, the reaction/gene is essential. Ratios of genes predicted by the model to be essential and non-essential were compared using Student *t*-tests. By measuring fluxes to biomass with a range of external metabolite fluxes (boundaries imposed on the exchange reactions) substrate compatibility was assessed. FVA was used to determine the range of flux for each reaction that were compatible with maximal growth under the given conditions.

### Data availability

The full array design is available in BμG@Sbase (BμG@Sbase: A-BUGS-30) [56] and also in ArrayExpress (ArrayExpress: A-BUGS-30). Fully annotated microarray data have been deposited in BμG@Sbase (accession number [E-BUGS-129]) [57] and also ArrayExpress (accession number [E-BUGS-129]). The genome-scale model (Nmb_iTM560) is available both as an Excel file (Additional file 1), and on the Surrey Genome Scale Metabolic Network modelling server [23], where it can either be used for direct online analysis (FBA, FVA, reaction and gene essentiality) or downloaded as a Cobra Toolbox compatible SBML. For further details please see the notes sheet in Additional file 1.

## Abbreviations

bp: base pairs; CAB: Columbia agar base; FBA: flux balance analysis; FVA: flux variability analysis; LPS: lipopolysaccaride; MM: minimal media; PEP: phosphoenolpyruvate; ROC: receiver operating characteristic; TCA: tricarboxylic acid; TraSH: transposon site hybridization; KDO: 2-keto-3-deoxyoctonoic acid.

## Authors' contributions

TAM conceived the study, carried out the experiments, carried out the bioinformatics analysis, constructed the model and was involved in drafting the manuscript. JN conceived the study and was involved in drafting the manuscript. JM conceived the study, constructed the model and was involved in drafting the manuscript. AMM carried out the bioinformatics analysis, constructed the model and was involved in drafting the manuscript. AMK carried out the bioinformatics analysis and constructed the model. All authors read and approved the final manuscript for publication.

## Supplementary Material

Additional file 1 **Nmb_iTM560 model**. An Excel file containing the Nmb_iTM560 model, metabolites, substrate conditions, stoichiometries and the original *i*AF1260 model of *E. coli *K12 [22]. Click here for file

Additional file 2 **Nmb_iTM560 biomass calculations**. An Excel file containing calculations to determine biomass components and stoichiometries, and molecular weights using data from the *i*AF1260 model of *E. coli *K12 [22] modified with *N. meningitidis*-specific data [19,25,26] Click here for file

Additional file 3 **Flux variable analysis**. An Excel file containing maximal and minimal reaction fluxes compatible with maximal flux to biomass were computed for minimal media agar and serum. Click here for file

Additional file 4 **Nmb_iTM560 predicted essential genes**. An Excel file containing lists of essential genes predicted by flux balance analysis. Individual genes were removed from the model and maximal possible growth rate determined. *In silico *gene knockouts that gave maximal fluxes to the biomass (the essential version) of approximately zero were considered essential. Click here for file

Additional file 5 **Microarray data and analysis for known mutants, assessment of bias in data and ROC analysis**. An Excel file containing TraSH data for mixes of known mutants, an assessment of bias within the TraSH data and receiver operating characteristic (ROC) analysis. Click here for file

Additional file 6 **Analyses of TraSH microarray data grouped by growth condition**. An Excel file containing TraSH data. Data are grouped by growth condition. Click here for file

Additional file 7 **Analyses of TraSH microarray data grouped by functionality**. An Excel file containing TraSH data. Data are grouped by functionality. Click here for file
